# “Orofacial Dystonia—A Silent Killer”: Mandibular Fractures with Orofacial Dystonia, A Report of a Case and Review

**DOI:** 10.1155/2021/6675961

**Published:** 2021-01-25

**Authors:** Anand deep Shukla, G. Srikanth, A. Chitra, Anupam Singh, Sunil Nayak

**Affiliations:** Department of Oral and Maxillofacial Surgery, MCODS, Manipal, India

## Abstract

Mandibular parasymphysis fracture is very commonly observed especially in old age when there is resorptions of the alveolar ridges. In cervical dystonia, there is centrally mediated disease in which there is uncontrolled and spasmodic contraction of the facial and the masticatory muscles. Due to the application of this sudden and uncontrolled force, there is a tendency of the bone to unfavourably remodel and weaken. The case presented here is of a geriatric patient who presented to us with a fracture at the right parasymphysis and left dentoalveolar region of the mandible and was suffering from cervical dystonia. Management of this case posed a challenge in every step, and it needed a resurgery where the fracture was managed by the placement of reconstruction plate. Not many cases in the literature have been reported where dystonic movements have resulted in the fracture of the mandible.

## 1. Introduction

Orofacial dystonia is a neuromuscular disorder of central origin which causes involuntary, spasmodic, and periodic movements of the muscles of the orofacial, masticatory, and lingual region [1].

Most of the cases are idiopathic (63%), other causes include drug induced (22.8), peripheral induced (9.3), post anoxic (2.55%), neurodegenerative disorder associated (1.8%), and head injury associated (0.8%) [2]. The triggering factors for this basically include physical or emotional stress, depression, or orofacial surgical procedures [3]. There is no clear-cut outline for the diagnosis and the management of this condition. The options mentioned in the literature for the management of dystonia include chemodenervation [4], medical management, and CNS procedures [5]. In the case that is presented, we came across a case of dystonia of long-term duration, which used to increase and decrease in intensity. The chronic dystonia led to the weakening of the mandible which resulted in the fracture of the parasymphysis region ultimately. There was also loosening of the teeth because of the dystonic movements. The case presented with challenge at each step.

## 2. Case Report

**Figure 1:**
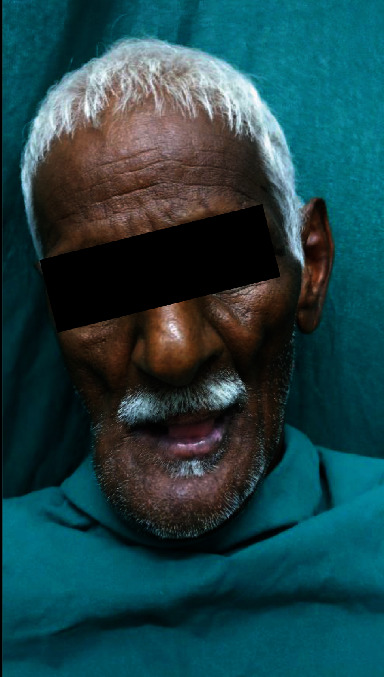
Preop photograph.

**Figure 2:**
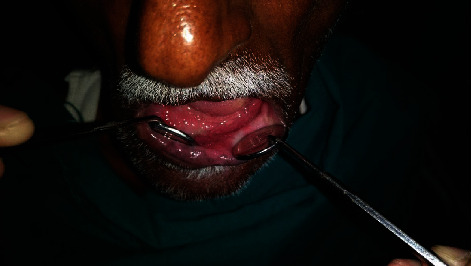
Intraoral view presop.

**Figure 3:**
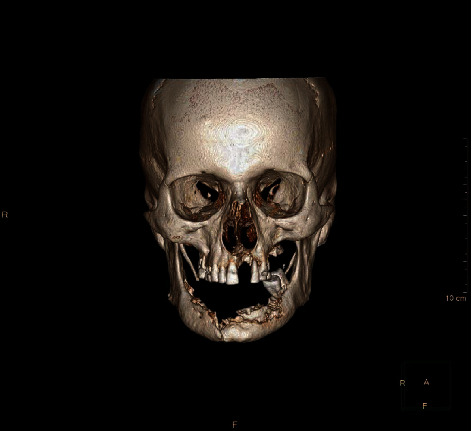
Preop CT scan showing right parasymphysis and dentoalveolar factures.

**Figure 4:**
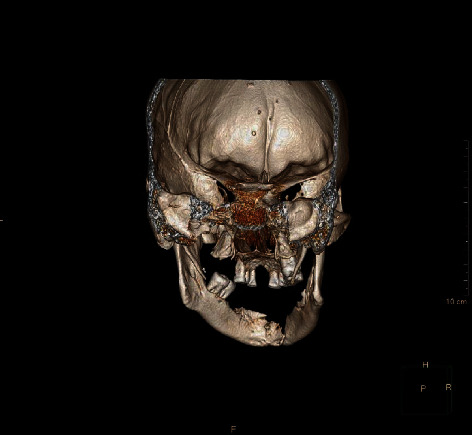
Lingual view of the same CT scan.

**Figure 5:**
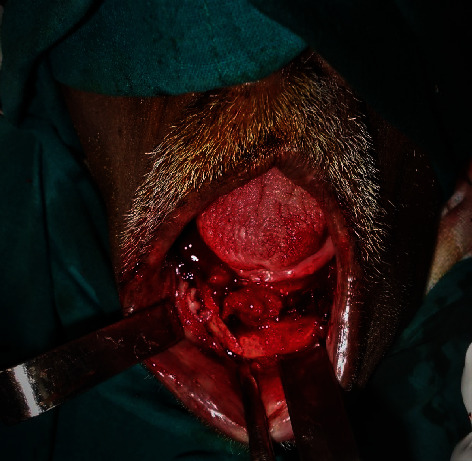
Coronal CT image.

This is a case of 56 years old male, who presented to us with the complaint of pain in the lower jaw since last one month ago (Figures 1 and 2). On thorough clinical and radiological examination, the patient was diagnosed with infected right mandibular parasymphysis and left dentoalveolar fracture (Figures 3, 4, and 5) and was admitted under our unit for the management of the same. The patient was also suffering from dystonia for the past one year. After clearances from the concerned units, the patient was taken up for surgery, where it was decided to do a thorough debridement of the infected area, send the bone for biopsy, and perform an open reduction and internal fixation of the fractured mandible.

**Figure 6:**
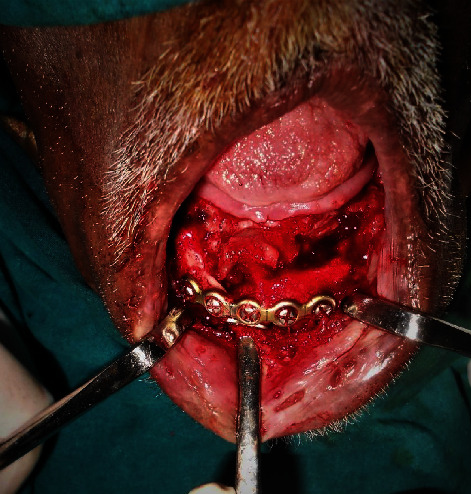
On-table exposure of the fracture site (first surgery).

**Figure 7:**
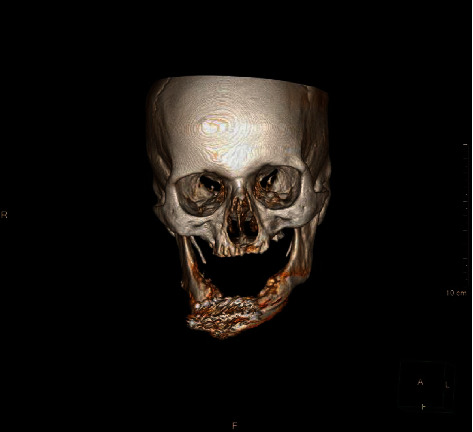
Plates in situ 9 (first surgery).

During the surgery, the mandibular parasymphysis and the dentoalveolar fracture were approached intraorally by giving a vestibular incision and performing layered dissection, after approaching the mandibular parasymphysis region, thorough debridement was done and the infected chunks of bone were removed. Following this, the fractures were fixed using two miniplates. Following the fixation, the incision site was thoroughly debrided and closed in layers (Figures 6 and 7).

Following the surgery, the patient was shifted to postop ICU and then to the ward.

On postop day one, the patient was having persistent cough in view of which blood and sputum culture was taken which revealed E. Faecalis sensitive to Linezolid and K. Pneumonia sensitive to Gentamycin. The patient was given IV Ampicillin, IV Gentamycin, and Tab Linezolid as advised by the medicine department. Neurology was consulted, and the same medication was continued. ENT consultation was sought in view of difficulty in speech for which conservative management was advised, and the patient recovered well post surgery. Malignancy and osteomyelitis were ruled out by histopathology report, and the fracture was attributed to the constant occlusal trauma due to cervical dystonia. Following this, the patient was discharged.

**Figure 8:**
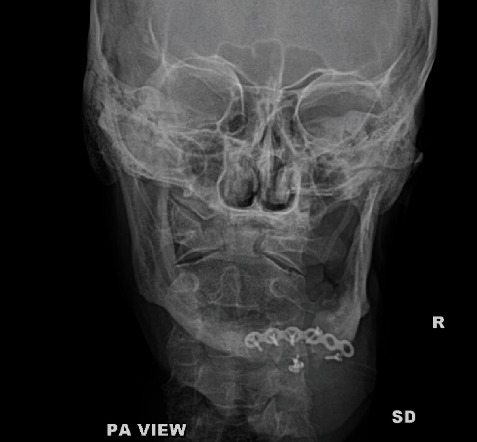
CT scan after hardware failure.

**Figure 9:**
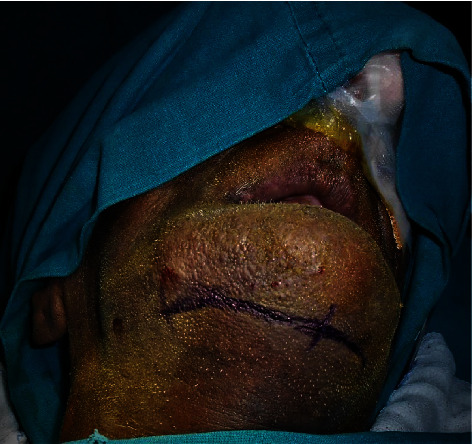
PA view of the mandible taken at the same time.

Following this surgery, the patient again reported to our outpatient department after three months with similar complaints of pain in the anterior region of the mandible. On examination, there was slight opening in the mucosa on the right parasymphysis region and segmental mobility over the right parasymphysis region. Clinical and radiological examination confirmed hardware failure to be the cause of pain and mobility over the right mandibular parasymphysis region (Figures 8 and 9).

**Figure 10:**
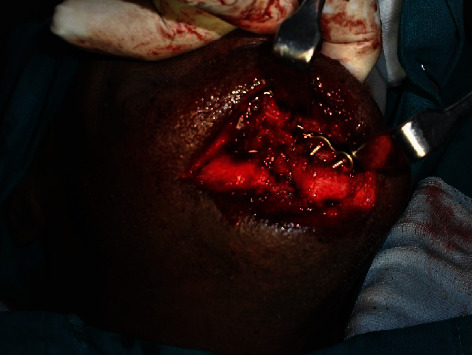
Extraoral markings for incision (second surgery).

**Figure 11:**
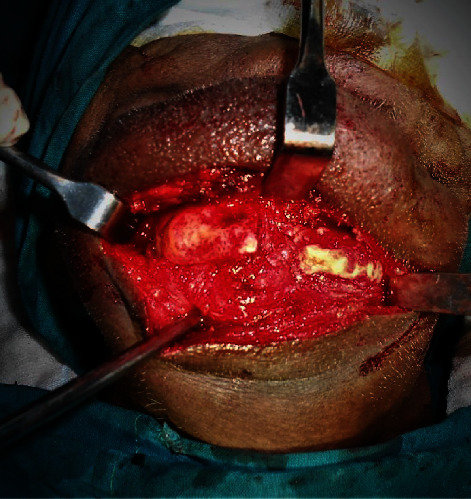
Exposure of the site, revealing a loose hardware.

**Figure 12:**
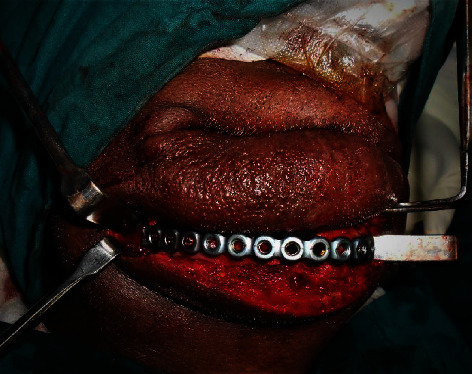
After the removal of the hardware and necrotic bone.

**Figure 13:**
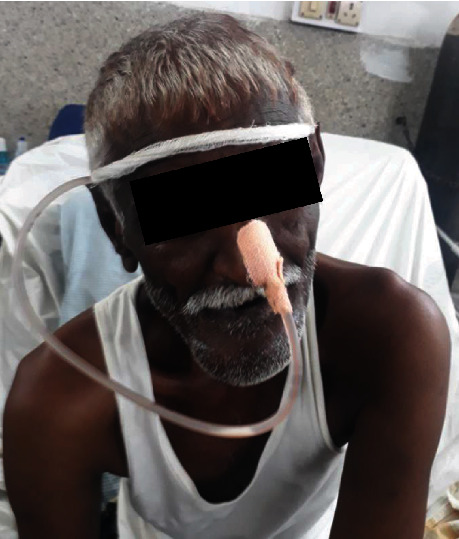
Plates in situ.

The patient was again taken up for the surgical intervention where after securing adequate anesthesia, extraoral incision from the right body region of the mandible to the left body region of the mandible was given. After layered dissection, the right mandibular parasymphysis region was exposed. On exposure, a dumbbell plate was found attached to the soft tissues with its two screws, which was subsequently retrieved (Figures 10 and 11). The superior 6-holed plate and 5 screws were also retrieved. Following this, the infected bone chunks were curetted out, and the mandible was fixed using a 15-hole 2.5 mm reconstruction cut plate and 8 (2 × 14 mm) locking screws (Figures 12 and 13). Homeostasis was achieved, and the wound was closed in layers.

**Figure 14:**
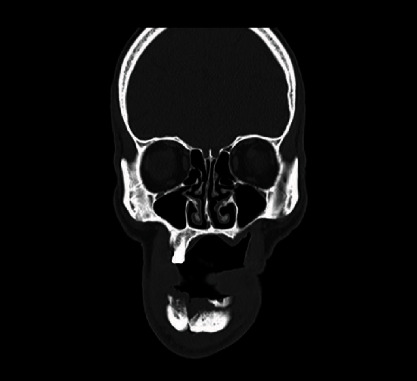
Postop photograph after second surgery.

The patient recovered well post surgery and was subsequently discharged after a week (Figure 14).

## 3. Discussion

Meige was the first person to describe orofacial dystonia in detail [6].This condition is also known by the name of Meige syndrome when it occurs in conjunction with blepharospasm [7]. This disease still poses a dilemma as far as its understanding is concerned. This condition may affect the muscles on one side or both the sides of the face, hence causing a different clinical presentation. All the jaw movements that include protrusion, retrusion, and side-to-side movements may be affected. When the muscles of facial expression and lingual musculature are affected, there may be spasmodic contraction; there are a number of features like twitching of the face, abnormal lip movements, nasal contractions, clenching/grinding of teeth, retractions of oral commissures, and/or abnormal movements of tongue [8].

There may be involvement of platysma also resulting in contractions of the neck. Involvement of laryngeal muscles has also been reported resulting in difficulty in breathing, dysphagia, dysphonia, and dysarthria [9].

Slowly, these uncontrolled spasms result in accumulation of products of anaerobic metabolism in the muscles and thus results in myalgia [10].

The pain further involves the antagonist muscles. On the other hand, spasms consistently lead to further damage to the soft tissue envelope. If the condition is not managed promptly, the condition becomes very serious resulting in intolerable pain and discomfort [11].

Stimulating factors for this episode are stress, depression, or any kind of activity involving the orofacial musculature. Even trauma has also been associated with the triggering of the episode of dystonia [12].

Over a period of time, the patient learns to live with the condition and they tend to deal with it by engaging in more pleasurable activities. The contraction of facial musculature gradually results in bruxism, clenching of the teeth, wearing away and loosening of the teeth, and even dislocation of the condyles [13]. In a few instances, it has also resulted in the fracture of the mandible due to excessive masticatory force generated, as was the presentation in our case.

Currently, there is no standard cure for dystonia, although various treatments have been documented. Treatment options for dystonia are as follows.

1. *Medical Management.* This includes administration of pharmacological agents like anticholinergics, anticonvulsants, antiparkinsonians, benzodiazepines, carbamezipine, lithium, and gabapentin, which are very effective in this condition [14]
2. *Chemodenervation.* It implies injections of botulinum neurotoxin in the form of Botulinum toxin A and B in focal dystonia
3. *Surgical Management including Peripheral/Central Nervous System Procedures.* This is used as the last retort when nothing is helping. Peripheral procedures include TMJ arthroscopy and surgery, myotomies, rhizectomies, and ramisectomies. CNS procedures involve deep brain stimulation or identification and ablation of a desired nucleus in the brain. Thalamotomy and pallidotomy are also reported to provide relief in some cases [15]

In our case, the patient was a 56-year-old male who was suffering from dystonia for the past 1.5 years. The patient came from a very low socioeconomic background and was not undergoing any treatment for cervical dystonia. The patient showed signs of twisting of the head and chin towards the side which was involuntary. He had difficulty in coordinating the movements of his jaw muscles. He was partially edentulous and was not using any dental prosthesis. When the patient presented to us, his chief complaint was the pain and tenderness over the right mandibular parasymphysis region; on examination and radiological examination, a diagnosis of fracture of the right mandibular parasymphysis region was made. There was no history of any trauma on that region. In all likelihood, the fracture had occurred some time back since there were obvious signs of infection evident. Since there was no history of trauma which was confirmed by the history taken from the patient as well as the relatives accompanying the patient, the most obvious cause of the fracture can be the occlusal forces due to dystonia resulting in the fracture of the mandibular parasymphysis. The patient also showed signs of bruxism which was confirmed by the presence of occlusal facets on the remainder of teeth. There were only two lower molar present which may have caused improper distribution of occlusal forces resulting in the fracture of the mandibular region. The patient and the patient's party gave the history that the dystonia that the patient was suffering from was idiopathic. He was also partially edentulous with loss of few anterior teeth and did not used a denture. Due to these factors, the occlusal load on the anterior mandible would have been increased multiple times leading to the eventual weakening of the region and leading to fracture of the anterior mandible. The primary surgery was aimed at thorough debridement of the infected bone, curettage, biopsy to know if it was a pathological fracture, and fixation of the mandible. The fixation that was done using two miniplates which were load sharing.

The biopsy report ruled out any pathological involvement in the form of malignancy or osteomyelitis. When the patient reported to us again, he had a hardware failure most probably due to dystonia and excessive masticatory forces. He was then taken up for surgery again, and the loosened hardware were removed, followed by fixation with a reconstruction plate, which is a load-bearing plate. The use of a heavy-load-bearing plate gives additional strength to the mandible and hence bears the excessive forces of mastication in dystonia patients. The patient recovered well after the surgery and had shown good improvement on his follow-up visits.

## 4. Conclusion

Orofacial dystonia is a very distressing condition which causes a lot of problems to the person concerned. Some general recommendations to be followed in these patients are as follows:

1. Periodic dental visits to look for health of the teeth and the surrounding structures
2. Periodic X-rays to be taken of the maxillofacial region, to rule out facial fractures or pathology caused due to dystonia
3. Good maintenance of oral hygiene
4. Proper administration of the medications for orofacial dystonia

If all these measures are undertaken and a periodic check-up, any problem arising can be diagnosed early and patients suffering can be reduced considerably.
